# Rate of recovery from perturbations as a means to forecast future stability of living systems

**DOI:** 10.1038/s41598-018-27573-0

**Published:** 2018-06-18

**Authors:** Amin Ghadami, Eleni Gourgou, Bogdan I. Epureanu

**Affiliations:** 1 Department of Mechanical Engineering, University of Michigan, Ann Arbor, Michigan 48109 USA; 2 Department of Internal Medicine, Medical School, University of Michigan, Ann Arbor, Michigan 48109 USA; 3 Present Address: Mechanical Engineering, University of Michigan, Ann Arbor, MI United States

## Abstract

Anticipating critical transitions in complex ecological and living systems is an important need because it is often difficult to restore a system to its pre-transition state once the transition occurs. Recent studies demonstrate that several indicators based on changes in ecological time series can indicate that the system is approaching an impending transition. An exciting question is, however, whether we can predict more characteristics of the future system stability using measurements taken away from the transition. We address this question by introducing a model-less forecasting method to forecast catastrophic transition of an experimental ecological system. The experiment is based on the dynamics of a yeast population, which is known to exhibit a catastrophic transition as the environment deteriorates. By measuring the system’s response to perturbations prior to transition, we forecast the distance to the upcoming transition, the type of the transition (i.e., catastrophic/non-catastrophic) and the future equilibrium points within a range near the transition. Experimental results suggest a strong potential for practical applicability of this approach for ecological systems which are at risk of catastrophic transitions, where there is a pressing need for information about upcoming thresholds.

## Introduction

**Figure 1:**
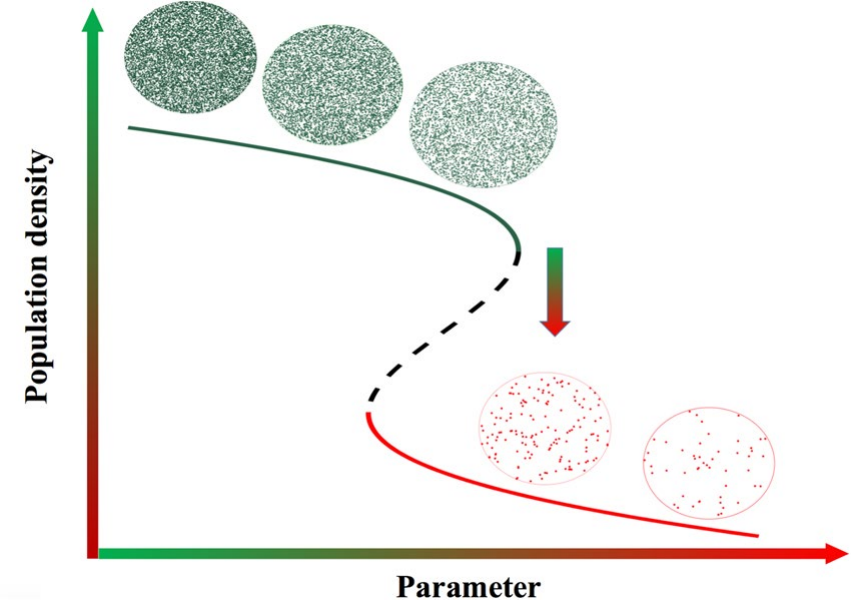
Schematic of a natural population subject to a critical transition. The state of the system changes abruptly from one equilibrium to another due to a slow change in the parameter.

Numerous studies have revealed a variety of systems that are at risk of undergoing critical transitions^1,2^, ranging from systems commonly examined in engineering^3,4^, chemistry^5,6^, physics^7,8^, and biology^9–11^, to others related to climate sciences^12–14^, medicine and disease^15–17^, social sciences^18–20^, and ecology^21–23^. Specifically, regime shifts in ecological systems have received growing attention as the cumulative human impact on the environment has increased the risk of ecological regime shifts^20–29^ (Fig. 1). Consequences include the collapse of natural populations which inhabit the ecosystem at risk^11^, and these consequences are important because typically it is exceedingly difficult to reverse the system to its pre-transition condition^30,31^ after a critical transition occurs. Hence, it is necessary to develop methods capable of forecasting the upcoming transition, as part of a preventive plan against possible detrimental consequences.

The need to forecast critical transitions for systems where a model is unavailable has resulted in the development of model-less approaches to extract early warning signals of such transitions from observed time series^32–40^. The advantage of applying early warning indicators to successfully raise the alarm when approaching a tipping point has been described in several recent studies^32–40^. However, many questions remain open. For example, although the indicators start to increase as the system approaches the tipping point, it is necessary to know quantitatively the distance (in the parameter space) to the upcoming transition to allow a better management of the system. Second, it is pivotal to know the type of the upcoming transition, i.e. whether the system is approaching a catastrophic or a non-catastrophic transition. However, based on nonlinear systems theory, critical slowing down^2,12,25,32,41^ and an increase in most available early warning indicators do not predict the type of transition, because they occur in both catastrophic and non-catastrophic transitions, such as transcritical and supercritical pitchfork bifurcations. In addition, management actions often require estimates for the future equilibrium points of the system before and after the transition. Such estimates have to be obtained using knowledge of the pre-transition system behavior. A forecasting method capable of addressing these questions would be of great importance for the management of ecosystems, as it would help to assess the existence of crucial thresholds and to evaluate the future consequences of surpassing them^42^.

**Figure 2:**
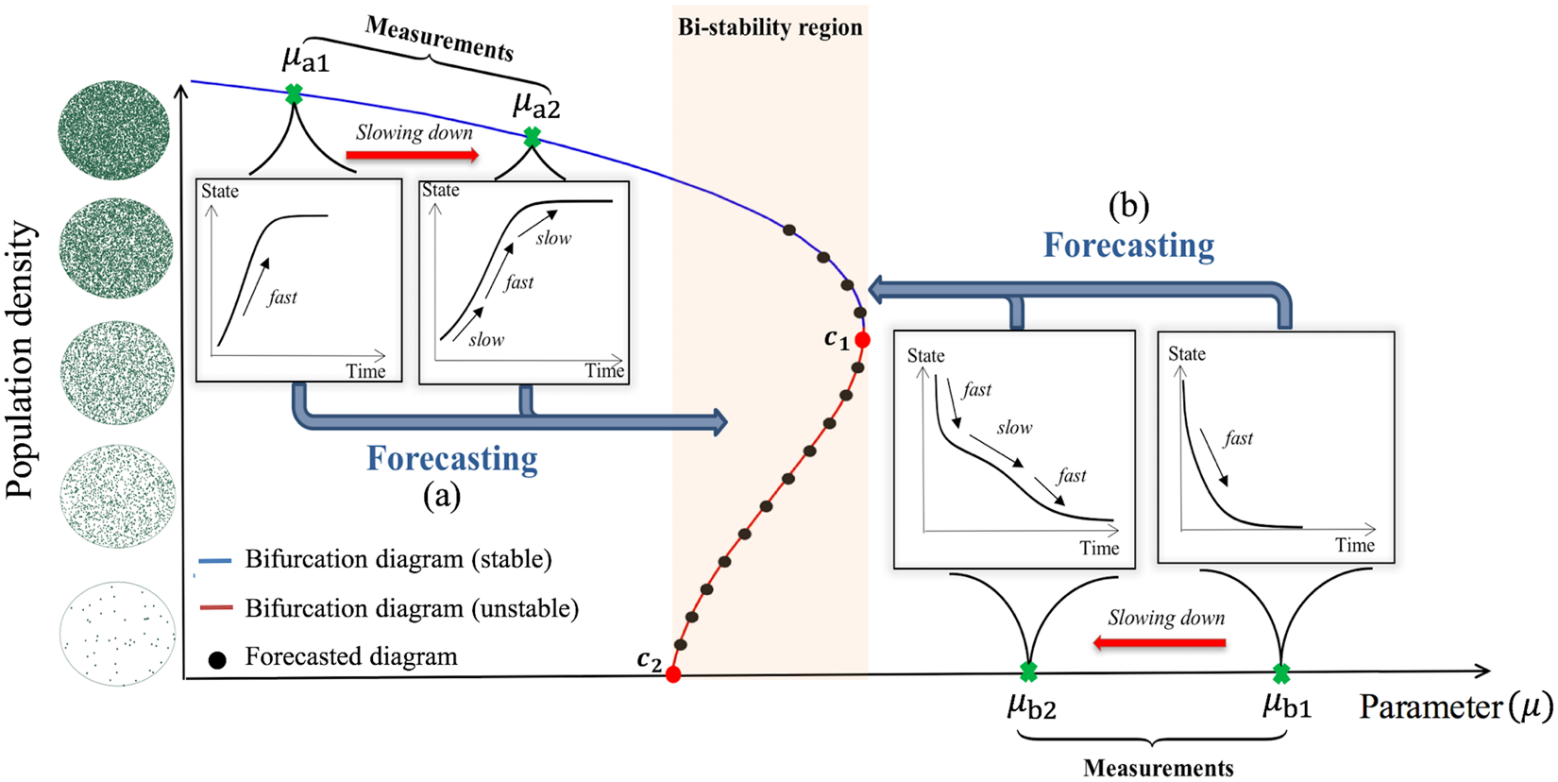
Schematic overview of the bifurcation forecasting method. Measurements from perturbations in pre-bifurcation conditions are employed to forecast the upcoming transition (black dots). As the system approaches a bifurcation, the rate of system’s recovery is decreased. At a fixed amplitude, the recovery rate decreases when the system approaches the bifurcation. At a fixed parameter, the smallest recovery rate corresponds to the amplitude which is closest to the actual bifurcation diagram. The future stability of the system is forecasted considering this change in the recovery rates. The system has two critical points (c_1_ and c_2_) which determine the boundaries of the bi-stability region. Cases (**a**,**b**) demonstrate a schematic of the forecasting procedure for the system operating at either side of the critical points, and are addressed in the experimental study.

In this paper, we introduce a model-less approach, referred to as the bifurcation forecasting method^4,8^, to address the aforementioned questions. Based on observations of the system response to perturbations only in the pre-transition regime, the method forecasts the bifurcation diagram which characterizes the stability and equilibria of the system in upcoming conditions (Fig. 2). The idea of the bifurcation forecasting method has been successfully evaluated using simulations and experiments in some engineered systems^3,4,8^. The aim of this paper is to introduce and investigate the application of the bifurcation forecasting method in living systems. The forecasting approach described herein uses the phenomenon of critical slowing down, namely the slowing down of the dynamics around an equilibrium when the system approaches a tipping point^41^. Note that recent observations have confirmed the existence of critical slowing down before tipping points in several natural ecosystems^43,44^.

Determining the conditions under which the forecasting method is reliable in ecological and biological systems requires experimental validation. To this end, we use a budding yeast population with cooperative growth, with sucrose as the carbon source, which is known to exhibit a critical transition as the environment deteriorates^11^. The existence of the Allee effect^45^ leads to bi-stability and a fold bifurcation at which the yeast population exhibits a catastrophic transition. This is accompanied by a critical slowing down phenomenon as the environment deteriorates, resembling an ecological collapse. The bifurcation parameter of the system is selected to be the mortality rate, which can be interpreted as a reflection of environmental conditions. As the mortality rate increases, the system is pushed toward a critical transition, resulting in population extinction.

The main focus of this study is to demonstrate and experimentally validate the suggested forecasting method, using a real living system. The forecasting method is also investigated theoretically, using the yeast population as a model system, as described in more detail in the Supplementary Information (SI).

## Results

**Figure 3:**
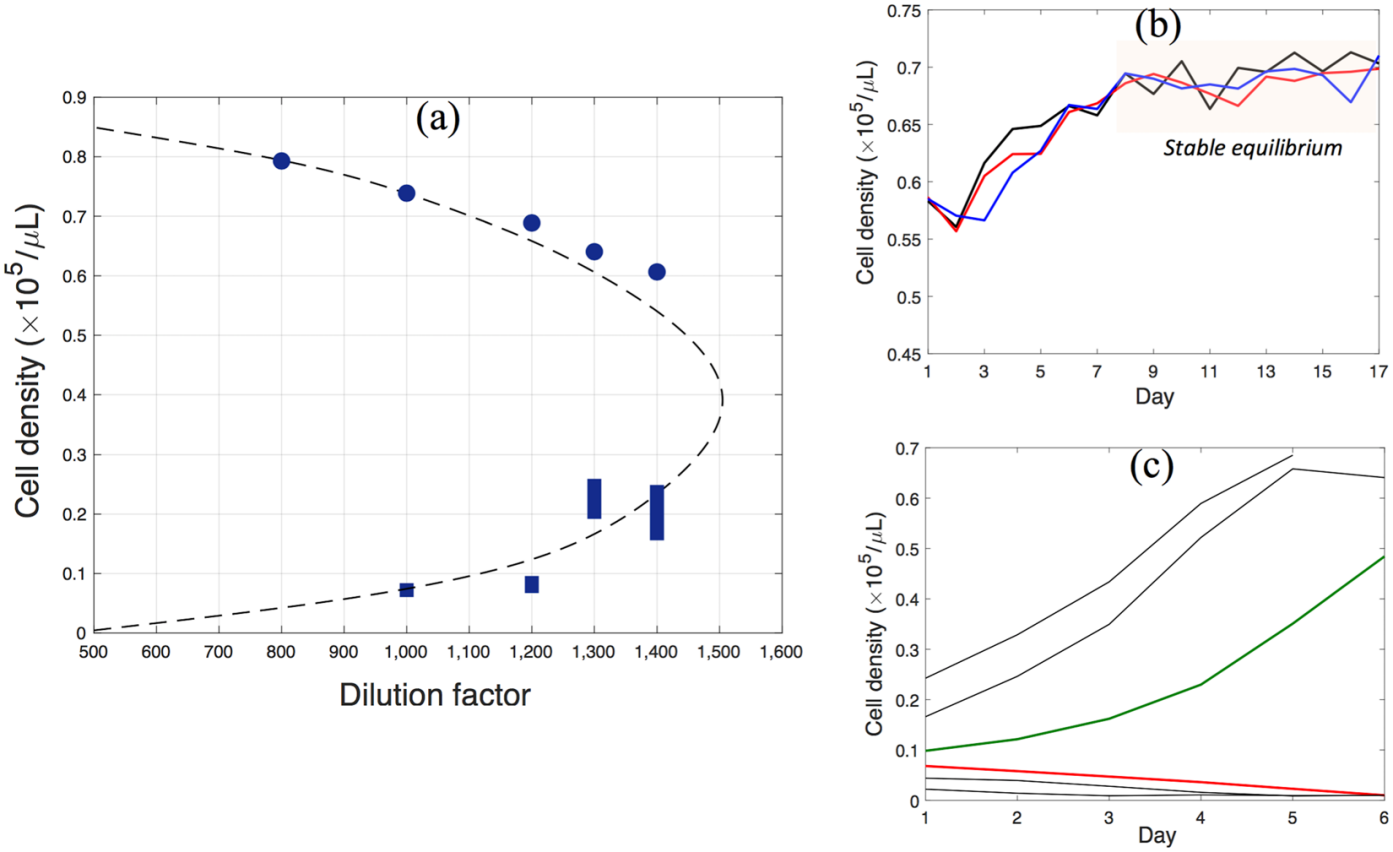
(**a**) Estimated bifurcation diagram of the buddying yeast system. Circles represent experimentally measured stable population densities at selected dilution factors obtained by averaging the populations which are at equilibrium over at least five days. Vertical bars represent experimentally approximated regions containing unstable fixed points at selected dilution factors, obtained by monitoring the population growth starting from several initial densities. Thus, the region between two initial conditions resulting in survival and extinction of the population is identified. (**b**,**c**) The experiments performed at the dilution factor of 1,200 to identify the corresponding stable and unstable fixed points on the bifurcation diagram are depicted. The dashed line is the approximated bifurcation diagram using the calibrated model. The dilution factor at the tipping point is approximately 1,505. This bifurcation diagram is used as a reference to validate the forecasting results presented in the following sections.

We monitor the effects of different dilution factors (mortality rates) on the density of the yeast population and on its ability to recover from perturbations. Figure 3 shows the approximated bifurcation diagram of the system based on experimental measurements (see also Section 2 of the SI). Experimental results show that the population becomes extinct at a dilution factor of 1,600, and is stable at a high density at a dilution factor of 1,400. Hence, the tipping point is expected to be between dilution factors 1,400 and 1,600. In general, the effects of noise, stochasticity, and the slowing down make it particularly challenging to measure stable and unstable fixed points close to the tipping point. The closer the system is to the tipping point, the worse the signal to noise ratio becomes, and the longer the system has to be observed to determine its stability. To address this challenge, we feed the experimental data into a genetic optimization algorithm to calibrate a simple two-phase growth model suggested by Dai and Gore^11^. The bifurcation diagram of the calibrated model which matches best the experimental observations is constructed, as depicted in Fig. 3. Based on the calibrated model, the dilution factor at the tipping point is estimated to be approximately 1,505. Although this approximation provides an estimation of the system stability, it contains uncertainty and hence may not necessarily represent the true dynamics of the system. Hence, the approximated bifurcation diagram using the model does not quite agree with the location of the experimental fixed points. Therefore, the mismatch between the forecasting results in the following sections and the model-based estimation of the bifurcation diagram is not solely due to the forecasting accuracy. Note also that this model-based estimation of the bifurcation diagram is used only as a reference to validate the forecasting results presented in the following sections; forecasting does not require any model of the system or any bifurcation diagram *a priori*.

We forecast the bifurcation diagram of the yeast population using measurements from one side of the bifurcation diagram. We demonstrate the forecasting method separately for each side (namely, see cases (a) and (b) in Fig. 2). Each case corresponds to a type of transition which is commonly reported for many systems. The first is the extinction case, where the population is approaching a critical transition which results in the collapse of the population, as is observed in ecological systems^1,30,41^ (case (a) in Fig. 2). The second is the emergence case, where a sudden increase in the population occurs, as is observed in outbreaks of infectious diseases^46–48^ (case (b) in Fig. 2).

**Figure 4:**
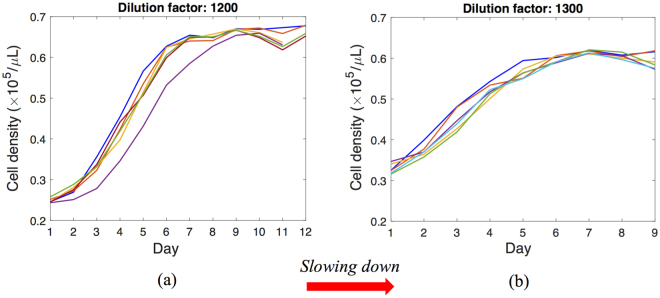
Measured recoveries of the yeast population from perturbations at: (**a**) a dilution factor 1,200; (**b**) a dilution factor 1,300, while the system approaches to the tipping point from parameter values smaller than the critical parameter. Each line represents a separate experiment, and each experiment has 7 replications which are performed simultaneously using the same source of materials at exactly the same experimental conditions. The system slows down while approaching the critical transition, namely as the dilution factor increases from 1,200 to 1,300 (changing from (**a**) to (**b**)). These measurements are used to forecast the bifurcation diagram which corresponds to the schematic shown in case (**a**) of Fig. 2.

As a first step we study the extinction case, which occurs as the dilution factor increases, and has values increasingly closer to the critical one (i.e. 1,505), as shown in case (a) in Fig. 2. The dilution factor is the bifurcation parameter, and the goal is to forecast both the type of upcoming transition and the dilution factor at which the extinction transition occurs. For the forecasting, we measure recoveries of the system from perturbations at two dilution factors (all prior to the transition). Specifically, we perturb the system using randomly selected initial population densities smaller than the stable values, at dilution factors of 1,200 and 1,300. Recoveries at other dilution factors can be also used. Figure 4 shows measured recoveries from perturbations.

**Figure 5:**
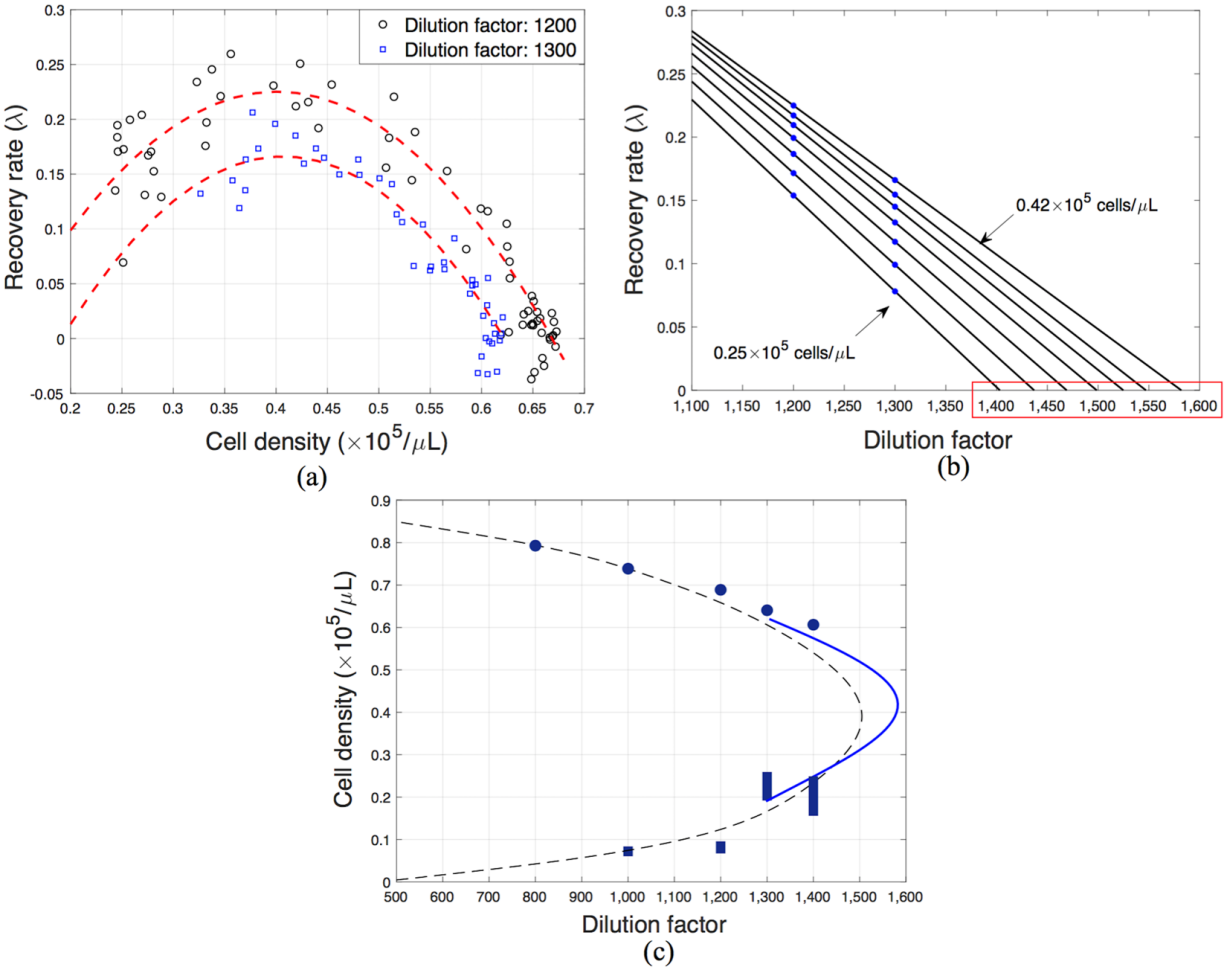
Forecasting using experimental measurements at dilution factors of 1,200 and 1,300, corresponding to case (**a**) shown in Fig. 2. (**a**) Recovery rates and quadratic polynomial fits at each amplitude estimated from the measurements shown in Fig. 5. (**b**) Example of extrapolated recovery rates at selected amplitudes to forecast the dilution factor at which the recovery rate is zero for each population density. (**c**) Forecasted bifurcation diagram (blue line). Circles and vertical bars represent experimentally approximated stable and unstable points on the bifurcation diagram, respectively. The dashed line is the approximated bifurcation diagram using the calibrated model.

Figure 5(a) shows the recovery rates estimated from measurements. To estimate the recovery rates, we use discrete approximations of the form ${\lambda }_{r}=(\mathrm{ln}\,{r}_{+}-\,\mathrm{ln}\,{r}_{-})/2$ at each measured population density, where *r*, *r*_−_ and *r*_+_ are the measured population densities at day *n*, *n*−1 and *n* + 1. The recovery rates are then smoothed using a second order polynomial that best fits the data (see Methods). As expected, results show that the recovery rates decrease at all densities as the system approaches the tipping point. More importantly, the recovery rate at each population density is correlated to its distance from the bifurcation diagram. The system has its maximum recovery rate at a population density around 0.400 × 10^5^ *cells*/*μL*, meaning that this density is the farthest point on the bifurcation diagram from the current dilution factor. Using the approximated recovery rates, one can extrapolate data in the *dilution factor - recovery rate* plane to approximate the dilution factor ($\tilde{\mu }$) at which the recovery rate for a specific density $(\tilde{r})$ is zero (Fig. 5(b)). The point $(\tilde{\mu },\tilde{r})$ is therefore a forecasted fixed point on the bifurcation diagram. This procedure is repeated for all available values of population densities, and the overall bifurcation diagram is forecasted (Fig. 5(c)). Based on the forecasting results, it is evident that the system is approaching a catastrophic fold bifurcation. Furthermore, the transition is forecasted to occur at a dilution factor of 1,582, where the population density is 0.420 × 10^5^ *cells*/*μL*. The upcoming stable and unstable fixed points after the critical transition are also forecasted.

Using such results, one can approximate the maximum perturbations that the system can tolerate in the upcoming environmental conditions. For instance, it is forecasted that at the dilution factor of 1,400, the population density would be stable at 0.575 × 10^5^ *cells*/*μL*, and if a perturbation pushes the population below the density of 0.248 × 10^5^ *cells*/*μL*, the population will not survive.

As a next step we study emergence, which occurs as the dilution factor decreases and has values closer and closer to the critical one (i.e., 1,505), as shown in case (b) in Fig. 2. In this case, the system has only one equilibrium (at zero density), and perturbations in the density cannot lead to a transition. In this case, the system does not exhibit a bifurcation; however, when the parameter decreases below the critical value, the system enters a region of bi-stability where a perturbation may push the system to a large non-zero equilibrium. Using measurements of the system behavior before entering the bi-stability region, the goals are (a) to forecast the parameter value at which the bi-stability region begins and (b) in general, to forecast the bifurcation diagram which identifies the type of upcoming transition and the system dynamics at upcoming parameter values. This type of forecasting might be of interest in many cases, as in ecological systems^49,50^, cancer dynamics^51,52^ and outbreaks of a disease^46–48^, where a system with a low density equilibrium may jump to a highly populated state.

In the experiments performed in this study, the critical dilution (and the beginning of the bi-stability region) is approximately 1,505 (Fig. 3). To forecast the upcoming bi-stability and possible transition, we monitor the population dynamics of buddying yeast in response to external perturbations, at three larger dilution factors, namely 1,800, 1,700 and 1,600.

**Figure 6:**
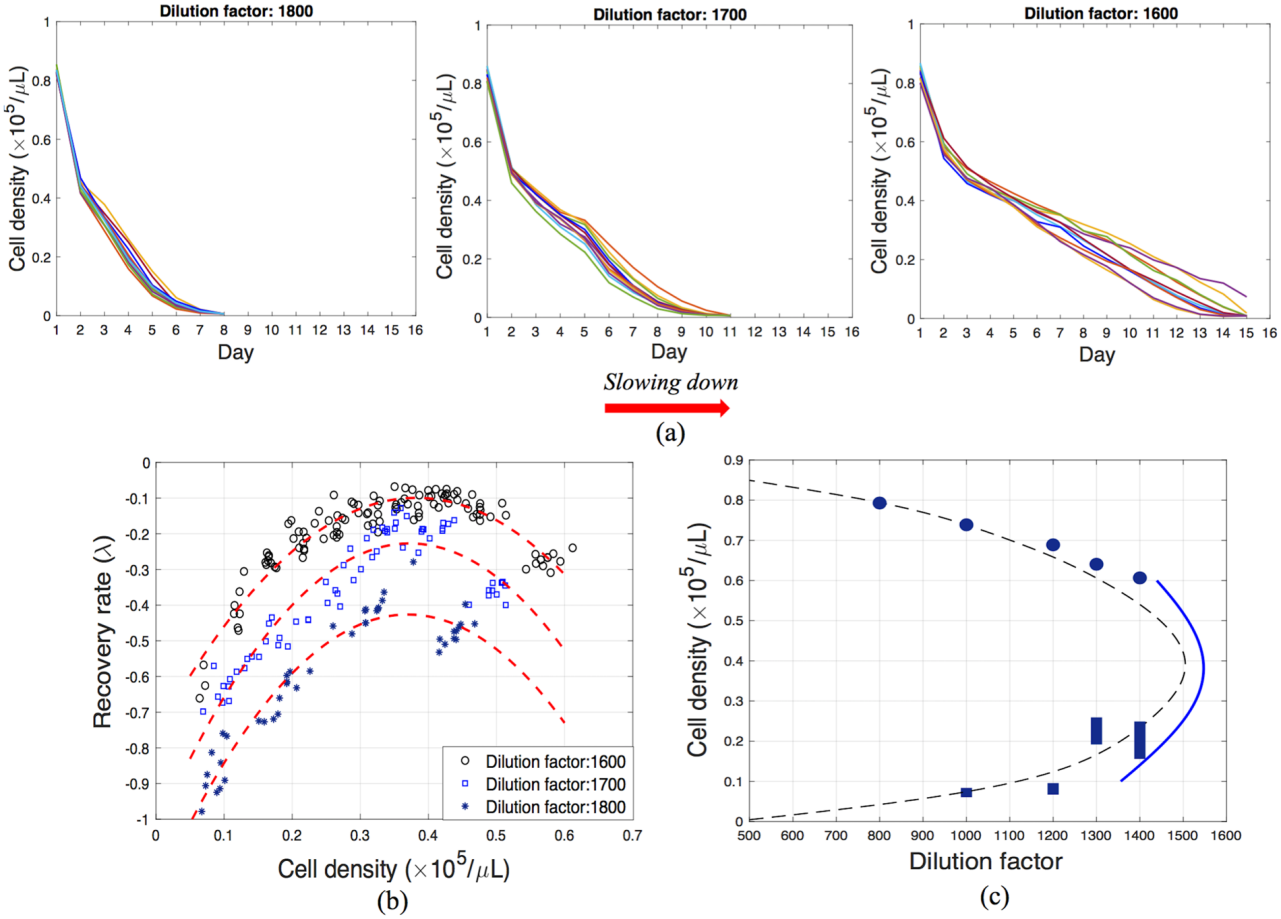
Forecasting using measurements at dilution factors of 1,600, 1,700 and 1,800 corresponding to case (**b**) shown in Fig. 2. (**a**) Measured recovery of the yeast population from perturbations at three selected dilution factors for the system approaching the tipping point from parameter values greater than the critical parameter. Each line represents a separate experiment, and each experiment has 7 replications which are performed simultaneously using the same source of materials at exactly the same experimental conditions. (**b**) Recovery rates at each amplitude computed from measurements shown in (**a**) and quadratic polynomial fits. (**c**) Forecasted bifurcation diagram (solid blue line). Circles and vertical solid bars represent experimentally approximated stable and unstable points on the bifurcation diagram. The dashed line is the approximated bifurcation diagram using the calibrated model.

The measured recoveries (Fig. 6(a)) show that the system undergoes slowing down as the dilution factor decreases from 1,800 to 1,600, namely it takes longer for the system to return to its equilibrium when the dilution factor decreases. Mathematically, this means that the recovery rate of the system (*λ*) decreases. The recovery rates are computed based on system responses and are shown in Fig. 6(b). The plots of recovery rates at all dilution factors exhibit a local maximum at the population density around 0.390 × 10^5^ *cells*/*μL*, which means that the system has the slowest recovery rate around this value. The forecasting method therefore predicts that the closest non-zero equilibrium has a density of 0.390 × 10^5^ *cells*/*μL*. Hence, we expect a jump in the population density to this value (or greater values) when the dilution factor is further decreased.

Using the approximated recovery rates, the bifurcation diagram is forecasted, as depicted in Fig. 6(c). Results predict that the system is approaching a fold (catastrophic) bifurcation, at the critical dilution factor of 1,547 where the critical population density is 0.390 × 10^5^ *cells*/*μL*, which lies within the expected range based on direct observations of the system. Furthermore, the population densities on the stable and unstable branches of the diagram are also approximated for a small region around the tipping point. The forecasting results predict that if the dilution factor is decreased to values smaller than 1,547, a large perturbation can change the system equilibrium from zero to a high population density.

## Discussion

Previous research has shown that several indicators based on changes in ecological time series can successfully ring the alarm when a system of interest approaches a critical transition. Our work highlights that by monitoring the system’s response to perturbations it is possible to gain even more information about the future stability of the system. The proposed method requires larger perturbations compared to the small fluctuations around the equilibrium state, which are required in early warning methods. However, we demonstrate that when such data can be obtained, the bifurcation diagram can be successfully extracted using the proposed forecasting method, leading to a deeper understanding of the system’s future stability.

Close to the bifurcation point, external perturbations lead to long transients before the system reaches its stable state, which means that the system’s recovery rate decreases. Therefore, the system’s recovery rate from perturbations can be used as an indicator, and is correlated to the distance to bifurcations. The idea of relating the system recovery from perturbations to the upcoming ecological transitions has been also applied in some recent studies^53,54^. Those studies demonstrate that the increase in total recovery time from a perturbation is a good indicator of approaching a tipping point. In contrast, here we show that examining individual recovery rates at each amplitude instead of the total recovery time reveals more information about the system stability, such as the distance (in the parameter space) to the tipping point, the type of the transition and future stable and unstable equilibria.

The experimental data presented in this study are in agreement with the forecasting results, despite the fact that no mathematical model was used for forecasting, and all measurements were performed prior to the transition. Also, an important finding of the forecasting method is that the points of the bifurcation diagram which are closer to the current state of the system are forecasted with more accuracy, compared to the rest of the diagram. For instance, in Fig. 6(c) the most accurately forecasted point is the tipping point, and the accuracy decreases for the points of the diagram that are farther. Similarly, it is expected that the forecasted points on the bifurcation diagram in Fig. 5(c) have the best accuracy around the dilution factor of 1,300, and the accuracy decreases toward the tipping point. Hence, one should account for this effect while interpreting forecasting results. Note that this variation in accuracy was expected based on the theory of the proposed forecasting method (see Methods), since the points on the bifurcation diagram are forecasted by extrapolating the recovery rates. Therefore, the highest accuracy corresponds to amplitudes with the recovery rates closest to zero, i.e. the amplitudes closest to the bifurcation.

The use of parameters close to the tipping point generates more accurate forecasting results, since a more pronounced slowing down is observed close to transitions. For systems with low noise, a few measurements at each parameter value are sufficient for an accurate forecasting. However, in systems strongly affected by measurement and/or process noise, the more measurements are obtained, the higher the forecasting accuracy becomes. Also, the larger the perturbation is, the larger the noise intensity can be for a similar accuracy. For example, the method loses accuracy when the noise intensity is very high and the perturbations are very small. In such cases, one may choose to evaluate the system’s stability using previously developed early warning signals^32–40^.

The perturbations required for forecasting do not need to have the same intensity or the same source. They could be caused by various sources, either natural or anthropogenic. However, depending on the source of the perturbations, each perturbation might create different amplitudes for the resulting (perturbed) dynamics. The amplitudes in the forecasted bifurcation diagram cannot exceed the amplitudes experienced by the system during these perturbations. Moreover, if perturbations are applied by an external intervention, one should be cautious of applying excessively large perturbations. Such perturbations might result in jumping to another equilibrium state, if the system is operating in the region of bi-stability. This is of higher importance for complex ecosystems, which contain several connected states. In that case, perturbing a single state might affect the dynamics of other states, as well as the ecosystem as a whole. Smaller perturbations can still be used to forecast the bifurcation diagram; however, the larger the perturbation is, the larger the region of the diagram that can be forecasted is.

The forecasting approach is advantageous for complex systems which are at risk of catastrophic transitions, where there is a pressing need for information about upcoming thresholds. This paper provides a tool to evaluate the stability of complex systems in more detail than early warning signals, and aids in the management of fragile ecosystems.

## Methods

### Forecasting method

The bifurcation forecasting method is a model-free approach based on time-series measurements of the system’s response to perturbations in the pre-transition regime^4,8^. The method uses the critical slowing down phenomenon, and is able to forecast bifurcation diagrams for systems exhibiting this phenomenon, such as systems approaching a fold, pitchfork, Hopf or transcritical bifurcation.

Consider a nonlinear system with a parameter *μ* and an amplitude *r*, where the amplitude represents the distance from the current state to the equilibrium state. The time rate of change of the amplitude during a recovery can be written as

1 $$\dot{r}=f(\mu ,r).$$

Since the amplitude can be large, we use a Taylor series only with respect to the parameter around the bifurcation point (*μ* = *μ*_*c*_). The equations governing the system can be expressed as

2 $$\dot{r}=r[p(r)+{\alpha }_{1}(r)(\mu -{\mu }_{c})+H.O.T.],$$

where *p*(*r*) and *α*_1_(*r*) are functions independent of the parameter *μ*, and *H.O.T*. represents higher order terms in (*μ* − *μ*_*c*_). Note that this relation means neither that the dynamics of the system have been linearized in state space, nor that the dynamics have small amplitudes. Therefore, the analysis is applied for large and small amplitudes near the critical value *μ*_*c*_, without being restricted to use small perturbations. This feature enables us to forecast a larger region of the bifurcation diagram, especially in subcritical bifurcations where the amplitude at a tipping point might be far away from the current equilibrium state. For instance, in a saddle node bifurcation, there is a tipping point which has large amplitude (point *c*_1_ in Fig. 2). Both the amplitude and the parameter at which this critical point occurs can be forecasted, an achievement of great importance since it marks the boundary of the bi-stability region.

The rate *λ*(*μ*, *r*) at which the system recovers to its equilibrium after a perturbation is defined as

3 $$\lambda (\mu ,r)=\frac{\dot{r}}{r}\cong \frac{\mathrm{ln}\,{r}_{+}-\,\mathrm{ln}\,{r}_{-}}{2{\rm{\Delta }}t},$$

where *Δt* is the time between samples, *r*_+_ is the value of the amplitude measured at time *t* + *Δt*, and *r*_−_ is the value of the amplitude measured at time *t* − *Δt*. The recovery rate is a function of both the system’s parameter and of the amplitude. For example, in an ecological system, the amplitude can be the population density and the parameter is an environmental feature, such as temperature, mortality rate or any other index representing a specific environmental condition.

The recovery rate plays the most important role in the forecasting method. The recovery rate is different at different amplitudes, and the rate of recovery at each amplitude cannot be compared with that at other amplitudes. At a fixed amplitude, the recovery rate decreases when a system approaches a bifurcation. Similarly, at a fixed parameter, the smallest recovery rate corresponds to the amplitude which is closest to the actual bifurcation diagram. This finding is used to predict the bifurcation diagram.

**Figure 7:**
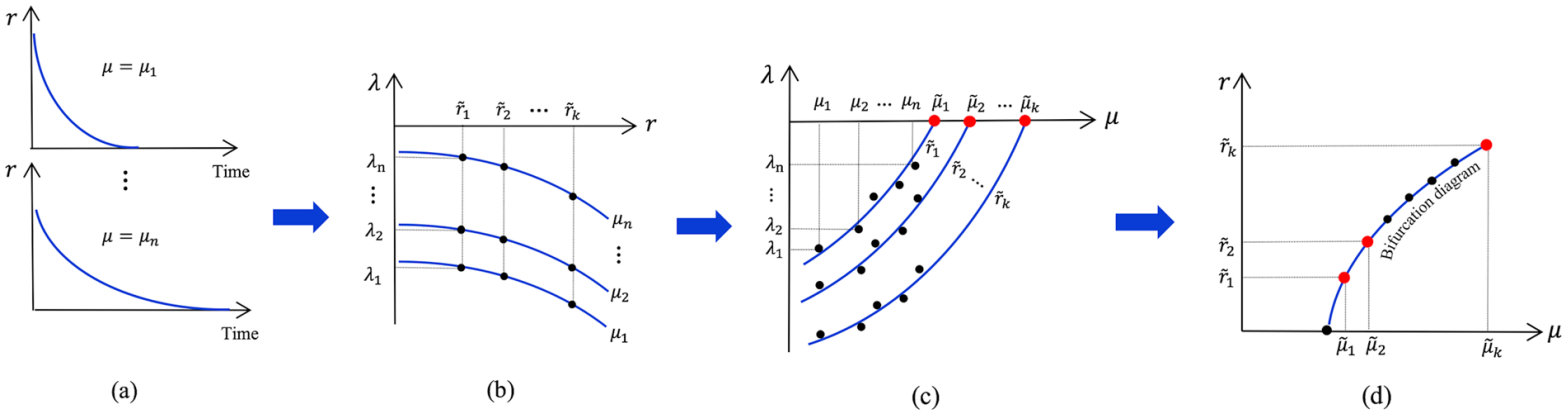
Steps followed to forecast the bifurcation diagram: (**a**) measure the system response to perturbations at several parameter values, (**b**) approximate the recovery rates from time series measurements, (**c**) identify the parameter $\tilde{\mu }$ at which the recovery rate for a specific amplitude $\tilde{r}$ is zero, (**d**) repeat steps (**b**,**c**) for several amplitudes $\tilde{r}$, and finally construct the bifurcation diagram using pairs of ($\tilde{\mu }$, $\tilde{r}$).

The schematic of the forecasting approach is demonstrated in Fig. 7. In this approach, we measure time series recoveries for several different parameter values *μ*_1_, *μ*_2_, …, *μ*_*n*_. At each amplitude $r=\tilde{r}$, the recovery rate is computed using a different set of measurements, i.e. $\lambda ({\mu }_{k},\tilde{r})$, $k=1,2,\,\ldots ,\,n$. Data in the $\mu -\lambda (\mu ,\tilde{r})$ plane are extrapolated based on Eq. (2) to identify the parameter $\mu =\tilde{\mu }$ at which the recovery rate is zero. The forecasted pair $(\tilde{\mu },\tilde{r})$ defines a fixed point at $\tilde{r}$ where the parameter value has the amplitude of $\tilde{\mu }$. This procedure can be repeated for different values of $\tilde{r}$, and thus the entire bifurcation diagram is forecasted (Fig. 7). Note that in the proposed approach, there is no difference between stable and unstable fixed points. Hence, the fixed points on the bifurcation diagram are forecasted regardless of their stability.

To forecast the bifurcation diagram, one needs measured recoveries at two or more parameter values in the pre-transition regime. In general, increasing the number of measured parameter values and the number of measured recoveries at each parameter value alleviates the effect of noise and enhances the forecasting accuracy. Note that by increasing the number of measured parameter values, one may include higher order terms in Eq. (2) to increase the forecasting accuracy, especially when the measurements are far from the bifurcation. However, it should be mentioned that increasing the order of extrapolation may also increase the impact of noise on the results, unless a large number of measurements is available. Furthermore, the computed recovery rates in practical applications are most probably noisy due to uncertainty in experiments (e.g., observe Figs 5(a) and 6(b) in the main text). To alleviate the effect of noise and stochasticity, we exploit the idea that for amplitudes close to a bifurcation point, the change in the recovery rates with amplitude can be approximated by a second order polynomial. We smoothen the measured recovery rates using a second order polynomial in the *amplitude* (*r*)*-recovery rate* (*λ*) plane (see Figs 5(a) and 6(b)). This idea comes from the observation that recovery rates correlate with the distance to fixed points on the bifurcation diagram, with a peak value at the critical amplitude (see the schematic in Fig. 2, and the recovery rates computed in Figs 5(a) and 6(b) for further clarification). This approximation holds for both supercritical and subcritical bifurcations, although the accuracy may vary for different systems.

There are a number of requirements for the forecasting method to be accurate. First, it is required that the system is close enough to the bifurcation to exhibit a measurable slowing down in the recorded recovery. The interpretation of “close enough” may vary for different systems, and depends on the system dynamics. Second, the obtained measurements have to contain identifiable parts on the inertial manifold to ensure that changes in the recovery rates are due to the slowing down phenomenon. The inertial manifold is an invariant set where the dynamics is the slowest in time and contains key dynamic features of the system^55,56^. This manifold is the slowest, and if the system starts from a state in this set, it remains in that set at all times. This fact needs more attention for large dimensional systems, where the fast dynamics of the system needs to be filtered out^3^. A third requirement is that the dynamics and the inertial manifold should vary smoothly with the bifurcation parameter. If the change in dynamics between two measured parameters is significant, the forecasting is not accurate^8^.

### Experimental procedure

The core design of our experiments is based on the system introduced by Dai and colleagues^11^, and used successfully in other experimental studies of catastrophic transitions and early warning signals^57–60^. The system is an ecological system designed by exploiting the cooperative growth of budding yeast in sucrose. The yeast system is used because it is easy to manipulate and displays key similarities with many natural populations.

We culture a selected yeast (*Saccharomyces cerevisiae*) strain under well-controlled conditions. The strain BY44741 is selected based on its auxotrophic properties and cooperative growth^61^. *Saccharomyces cerevisiae* displays cooperative growth, where individual cells use metabolites produced extracellularly by neighbor cells^58^. Hence, the system possesses a strong Allee effect^45^, which can cause bi-stability and lead to catastrophic transition. The cell density is an important factor affecting population growth in this system. On one hand, at low densities it is difficult for cells to cooperate and produce enough resources to survive. On the other hand, the resources are not enough at high densities, and the growth rate is decreased due to competition among cells. Therefore, at intermediate densities, the *per capita* growth rate is maximal, while it is negative at low and high densities^11^.

All experiments were performed on 96-well flat bottom sterile polystyrene microplates, Corning Falcon, USA, at 200 uL volume, in an IncuShaker orbital shaker, Denville Scientific, USA, at 30 °*C*, 300 *rpm*, in synthetic growth medium of BD Difco Dehydrated Culture Media Yeast Nitrogen Base (YNB) without amino acids, with the addition of Complete Supplement Mixture (CSM), Sunrise Science, USA, and 2% sucrose made from 20% sucrose stock solution in 1 *mM* Tris buffer, pH 8.0, UV and filter sterilized, added fresh daily to the YNB/CSM mixture. All experiments in the same series were performed with the same solutions, to avoid variations in the experimental system. The bifurcation parameter of the system was selected to be the mortality rate, introduced to the system by dilution. Namely, only a fraction of the population was transferred into fresh media every day. This corresponds to a population that is affected by an incident occurring periodically, such as invasion or the breakout of a disease, at which only a fraction of the initial population survives. Dilutions were performed every 23 hours, in predefined dilution factors, after the population density was monitored in a Cytation 1 Cell Imaging Multi-Mode Reader, BioTek, USA, by measuring the optical density at 620 *nm*. For each dilution factor, we performed 6 to 10 parallel experiments. Furthermore, each experiment had 7 replications using the same source of materials at the same experimental conditions. To this end, we prepared every day the initial sample and distributed it into 7 wells of a 96-well microplate, 200 *μL* each. Perturbations were applied to the system using randomly selected initial population densities, and the recovery from perturbations was measured until the population returned to its equilibrium state.

At low mortality rates and after a perturbation, the population recovers to its non-zero equilibrium with a high recovery rate. Furthermore, the population can survive from large perturbations since the cooperative growth of cells dominates the effect of mortality on the system. As the mortality rate increases, the system is pushed toward a critical transition resulting in population extinction. The reverse occurs as the mortality decreases, leading to emergence.

### Data availability

The experimental data supporting this study are available upon reasonable request.

## Electronic supplementary material

Supplementary Information
