# Supplementary material for: Rate of recovery from perturbations as a means to forecast future stability of living systems
Source: Sci Rep. 2018 Jun 18;8:9271. doi: 10.1038/s41598-018-27573-0 (PMC6006279; doi:10.1038/s41598-018-27573-0)
Supplement: Supplementary file 1 — Supplementary Information [file 41598_2018_27573_MOESM1_ESM.pdf]

Supplementary Information

Rate of recovery from perturbations as a means to forecast future stability of living systems

Amin Ghadami[1], Eleni Gourgou[1,2,a], Bogdan I. Epureanu[1,b]

[1] *Department of Mechanical Engineering, University of Michigan, Ann Arbor, Michigan 48109, USA*

[2] *Department of Internal Medicine, Medical School, University of Michigan, Ann Arbor, Michigan 48109, USA*

[a] current address: Mechanical Engineering, University of Michigan, Ann Arbor, MI, United States
[b] epureanu@umich.edu (the corresponding author)

1. Calibration curve

Cell density was tracked by measuring the optical density of the yeast population at $620nm$. A calibration curve was constructed, showing the corresponding cell density for every measured optical density. To this aim, a series of yeast samples with different densities were prepared, at $200\mu L$ final volume. The optical density of each sample was measured using the Cytation 1 Cell Imaging Multi-Mode Reader, BioTek, USA. Next, the actual cell density of each sample was measured using the Scepter 2.0 Handheld Automated Cell Counter, EMD Millipore, USA. Measured values are shown in Fig. S1 by square symbols. A third order polynomial is fitted to the data and used as the calibration curve in the performed data analysis.

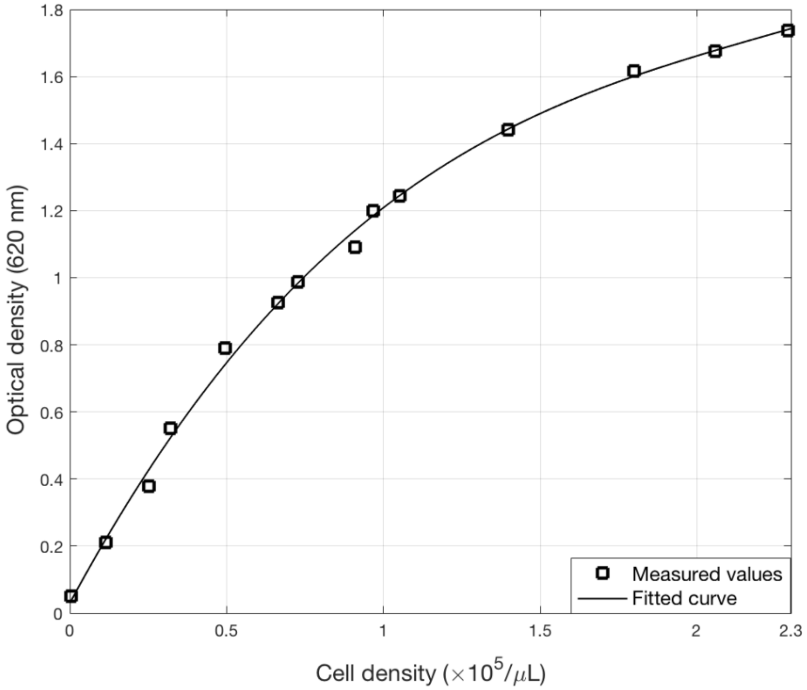

Fig. S1. Calibration curve mapping the measured $620nm$ optical density to the actual cell density.

2. Experimental points on the bifurcation diagram

A series of experiments were performed to identify several stable and unstable fixed points on the bifurcation diagram. We selected the dilution factors of 800, $1{,}000$, $1{,}200$, $1{,}300$ and $1{,}400$ to identify the non-zero stable values of population density at these parameter values. For this purpose, we started the experiments from selected initial population densities, and the system was monitored until the populations reached and stayed at their equilibrium state for several days. The stable values on the diagram were obtained by averaging the populations over the period during which they remained at their equilibrium state (Fig. S2).

Approximating unstable fixed points is more challenging since the system diverges from them. To approximate the values on the unstable branch, we considered dilution factors of $1{,}000$, $1{,}200$, $1{,}300$ and $1{,}400$. At each dilution factor, we monitored the population growth starting from several initial conditions. The region between two initial conditions resulting one in survival and one in extinction of the population was selected as the region containing the unstable fixed point (Fig. S3). Although the value of the unstable fixed points is not determined with high accuracy, this procedure provides an acceptable approximation of the unstable branch. This is required in order to evaluate the forecasting results, as well as to conduct the model calibration described in the next section.

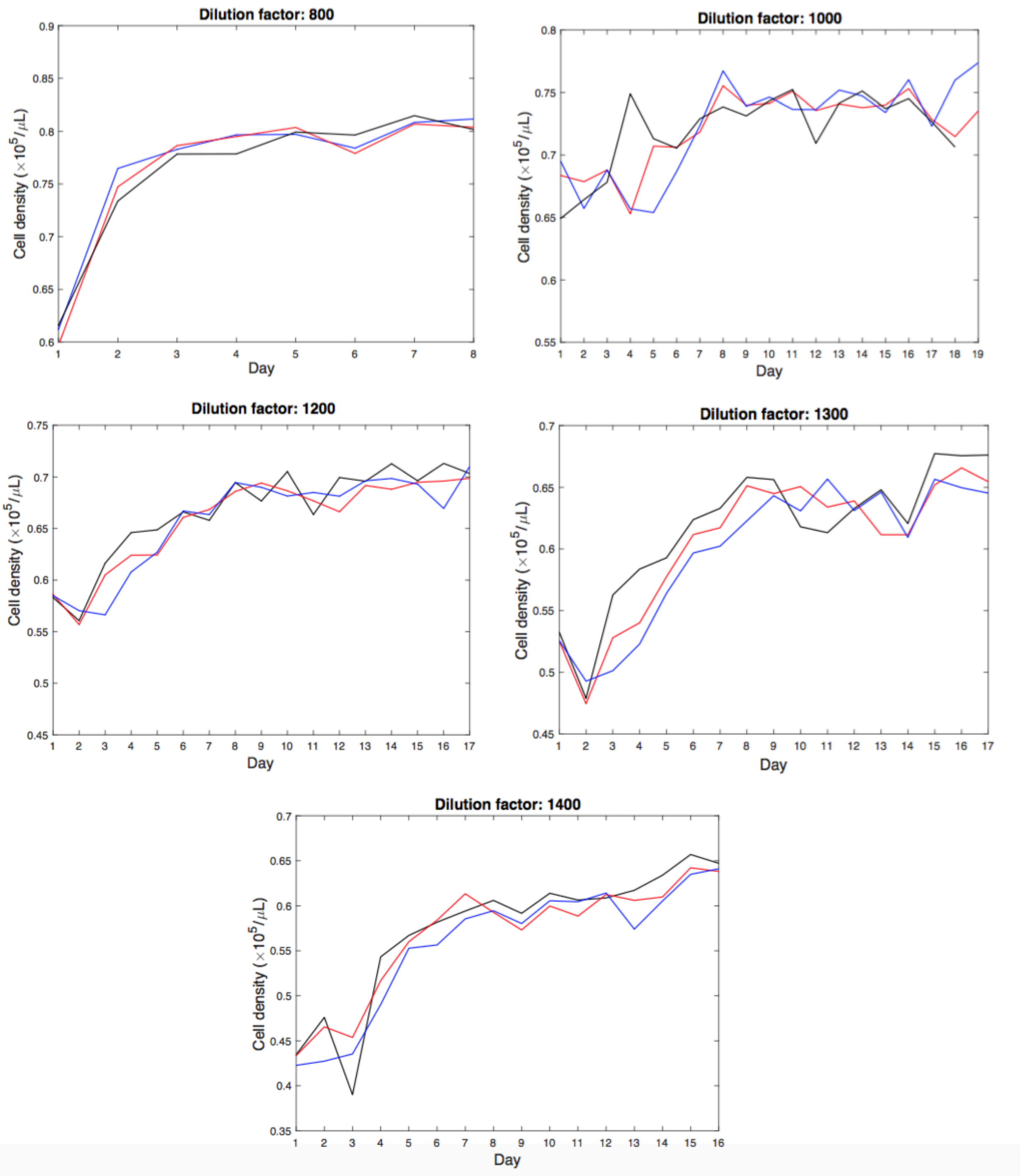

Fig. S2. Results of the experiments performed to identify the stable fixed points of the bifurcation diagram. Three sets of experiments are run at each dilution factor. The stable values at each dilution factor are approximated by averaging the populations over the period during which they remain at the equilibrium state.

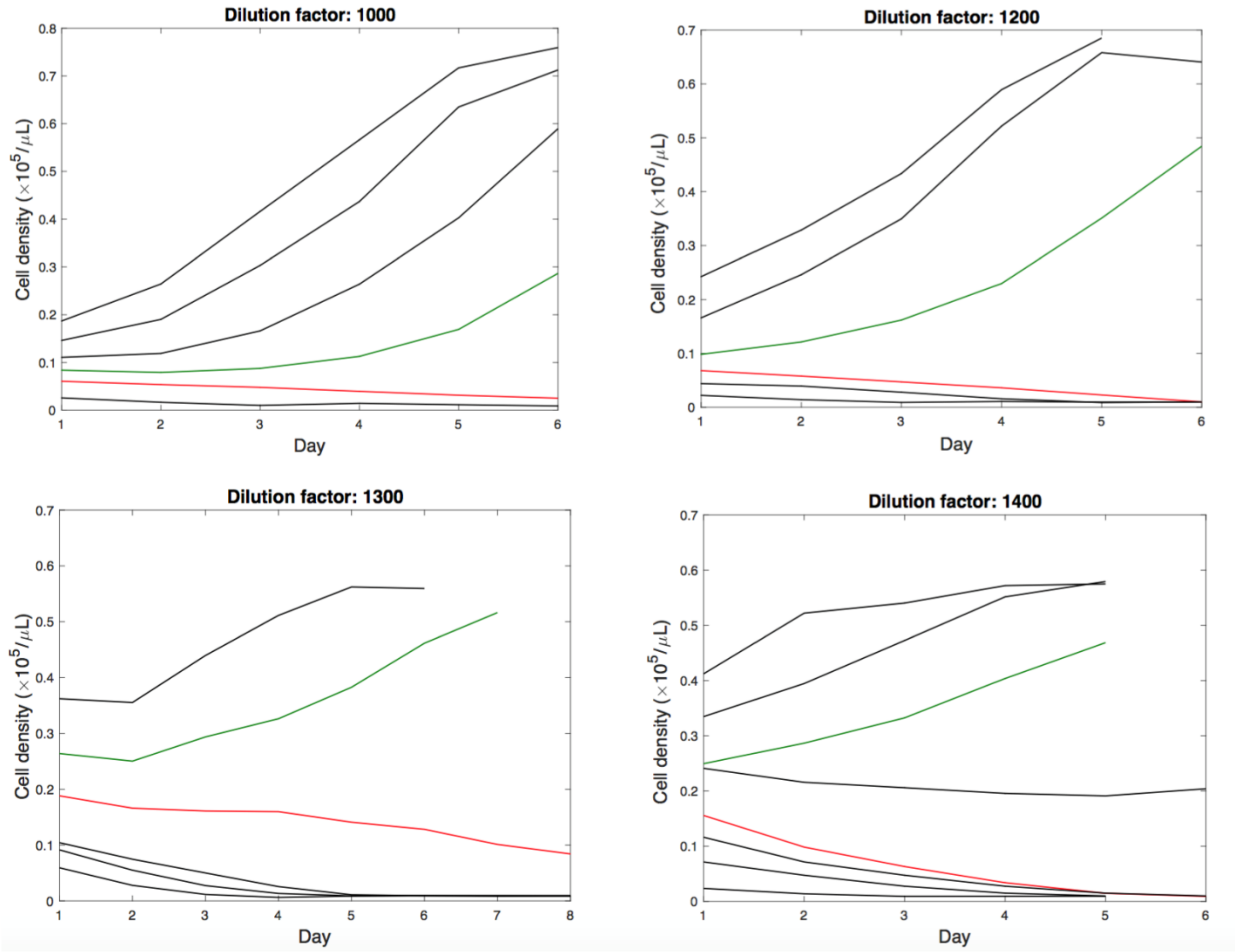

Fig. S3. Results of the experiments performed to identify the unstable fixed points of the bifurcation diagram. At each dilution factor, the region between two initial conditions resulting in survival and extinction of the population is selected as the region containing the unstable fixed point.

3. Model calibration not used for forecasting

The forecasting method is a model-less approach, suitable for systems for which no mathematical model is available. However, based on the model suggested for the yeast growth by Dai et al.[1], we use the experimental data to find the best model parameters which match our experiments. This is done for two reasons. First, we build a model as close as possible to the experimental data to show that the proposed forecasting method can be successfully applied when no measurement or process noise is available in the system. Second, measuring stable and unstable fixed points close to the tipping point is practically not feasible in experiments due to noise and

stochasticity in the dynamics. Since the focus of the proposed method is on the forecasting of tipping points and the parameter values close to these points, approximating the bifurcation diagram theoretically helps better interpret the results.

The two-phase mathematical model is in the following form[1]:

$$\frac{1}{N}\frac{dN}{dt} = \begin{cases} \gamma_{low} & 0 < N < N_c \\ \gamma_{high}\left(1 - \frac{N}{K}\right) & N_c < N < K \end{cases}, \tag{S1}$$

where N is the population density, K is the carrying capacity, γ_{low} and γ_{high} are the per capita growth rates for population densities smaller and greater than a threshold N_c. Moreover, there is a lag before the cells start to grow after they are transferred to a new media. In the simulations, this time lag is included in the 23hrs time period which is considered for the cells' growth.

To approximate the five unknown parameters of this model, we fed the experimental measurements of the bifurcation diagram into a genetic algorithm, and the model parameters best matching the experiments were extracted as shown in Table S1.

Table S1. Calibrated model parameters using experimental data and a genetic algorithm

	$\boldsymbol{K}$ $Cells/\mu L$	$\boldsymbol{\gamma_{low}}$ (hr^{-1})	$\boldsymbol{\gamma_{high}}$ (hr^{-1})	$\boldsymbol{N_c}$ $(Cells/\mu L)$	$\boldsymbol{Time\ Lag}$ (hr)
**Model fitting**	0.923×10^5	0.255	0.419	174	1.38
**Experiment**	0.854×10^5	No measurements	0.503	No measurements	1.33

We have also computed the three parameters $\boldsymbol{K}$, γ_{high} and the time lag experimentally to evaluate the validity of the genetic algorithm results. To approximate the time lag before population growth, several experiments were initiated starting from different initial conditions. We started recording the population growth right after the initiation of the experiment using the plate reader. The plate was placed in the plate reader, with the temperature set to $30°C$ and the orbital shaking speed at

$300rpm$. The optical density was measured every 10min for a total of 4.5hrs. The results depicted in Fig. S4 show that the population starts an exponential growth after approximately 80min, which represents the experimentally defined time lag.

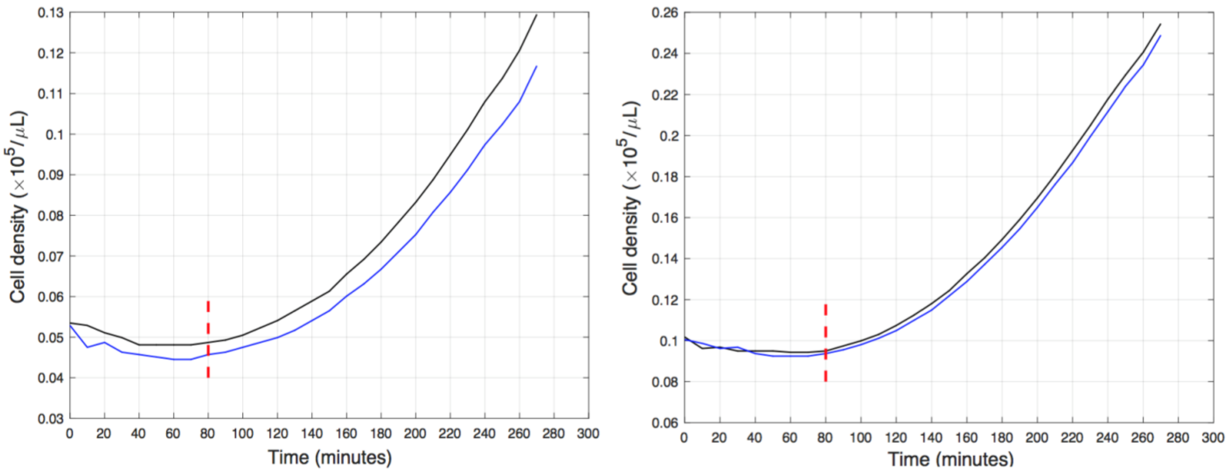

Fig. S4. Estimating the time lag before population growth. Experiments are initiated from 2 sets of different initial conditions, and the population growth is monitored for 4.5hrs. Each solid line represents a separate experiment, and the estimated time lag is marked by a dashed line on each figure.

To approximate γ_{high} and K, we monitored the population growth starting from an initial density of 0.188×10^5 $cells/\mu L$, which is expected to be greater than N_c. The experiment was initiated several hours in advance to make sure that the population was in the exponential growth phase (log phase). The population was then monitored for 17hrs until it reached and stayed at its saturated state (stationary phase) for about 5hrs (Fig. S5). We prepared the initial sample in a large volume and aliquoted the homogenous solution into 48 wells of a 96-well microplate, $200\mu L$ each. Results are shown in Fig. S5. Considering a logistic growth for the population, the parameters are found to be $\gamma_{high} \approx 0.503\ hr^{-1}$ and $K \approx 0.854\times10^5\ cells/\mu L$.

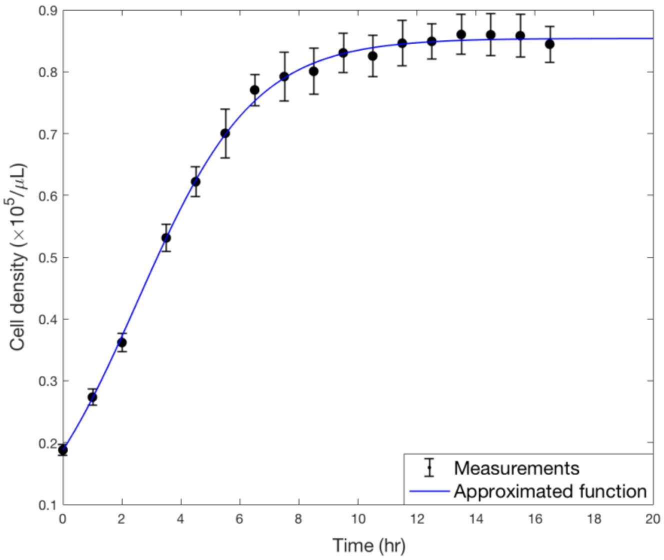

Fig. S5. Experimental results of estimating γ_{high} and K. A population started from intermediate initial density was observed over 17hrs. Experimental results are demonstrated by mean and standard deviation error bars of 48 simultaneously performed replications.

Comparing the directly measured parameters to those obtained by using the genetic algorithm (in Table S1), it is observed that they are in the same range.

The calibrated model is used to generate the bifurcation diagram of the system (Fig. 3) as a reference to be compared with the forecasting results.

4. Simple theoretical examples

In this section, we apply the method to forecast the bifurcation diagrams of simple systems exhibiting pitchfork bifurcations. We consider both supercritical (non-catastrophic) and subcritical (catastrophic) bifurcations to verify that the approach can differentiate between these two cases. Thus, we consider the following dynamical systems, where r is the amplitude and μ is the parameter:

System 1 : $\dot{r} = \mu r - r^3,$ (S2)

System 2 : $\dot{r} = \mu r - r^3 + r^5$, (S3)

where “System 1” and “System 2” exhibit supercritical (non-catastrophic) and subcritical (catastrophic) transitions, respectively. The systems are perturbed at two parameter values before their corresponding critical points, and the forecasting method is applied to estimate the bifurcation diagrams and identify the type of the upcoming transition. The forecasting procedure and results are shown in Figs. S6 and S7. These two figures show that the method successfully forecasts the bifurcation diagrams and tipping points of both examined systems. Moreover, one can observe from the forecasted diagrams that “System 1” is approaching a non-catastrophic transition, while the transition in “System 2” is catastrophic.

Comparing the plots of recovery rates of the systems reveals the difference between the rate of system recoveries from perturbations in catastrophic and non-catastrophic transition. In “System 1”, the slowest recovery rate occurs at zero amplitude. However, in “System 2”, the slowest recovery rate corresponds to non-zero amplitude. This amplitude corresponds to the saddle node bifurcation point (i.e., the turning point), which is a feature of catastrophic transitions. The amplitude which exhibits the slowest recovery rate is the one which is closest to the bifurcation diagram. This fact can be observed comparing the recovery rate plots and the forecasted bifurcation diagrams in Figs S6 and S7.

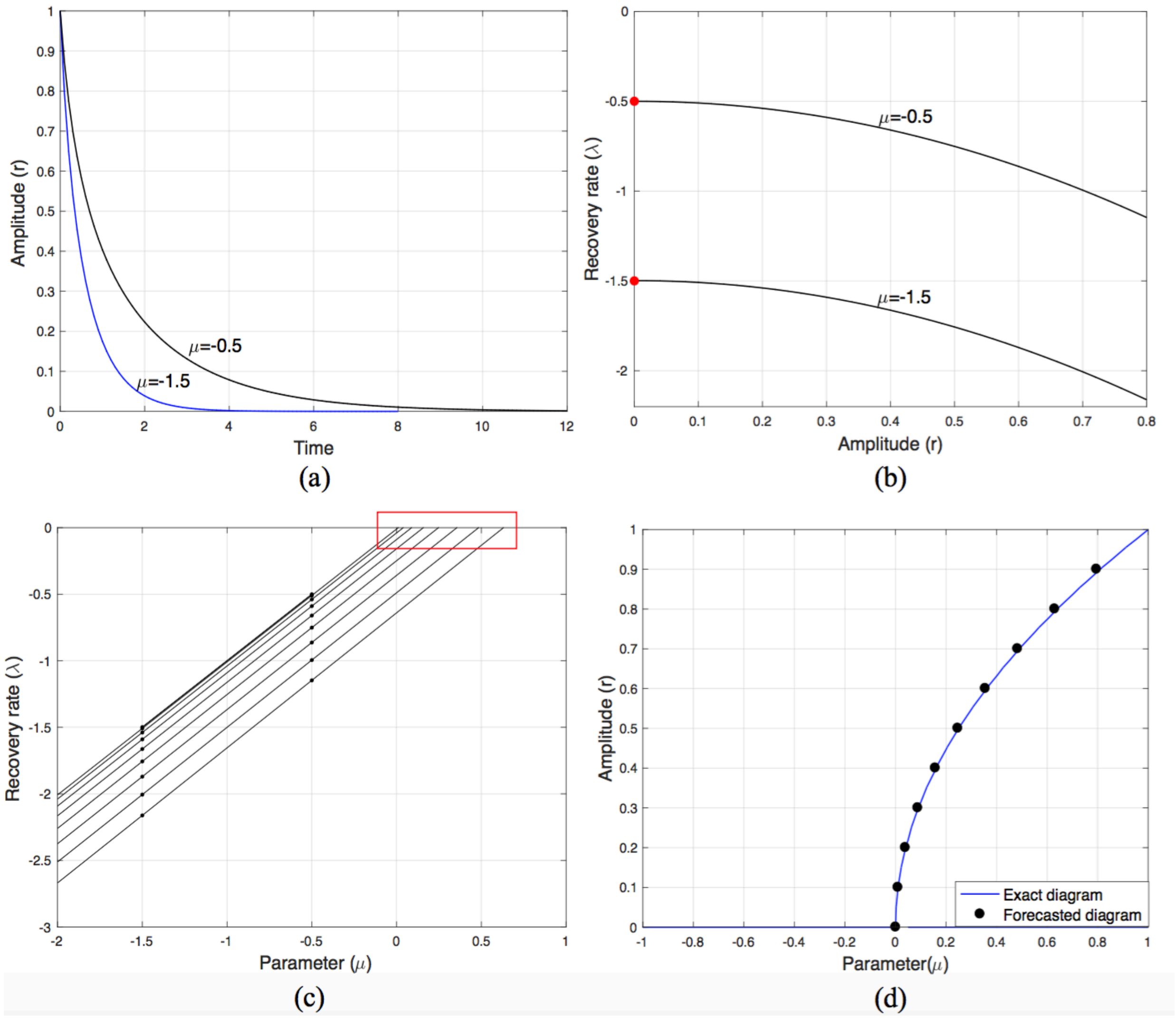

Fig. S6. Simulation results and forecasting procedure of "System 1" governed by Eq. (S5) and exhibiting a supercritical (non-catastrophic) transition.

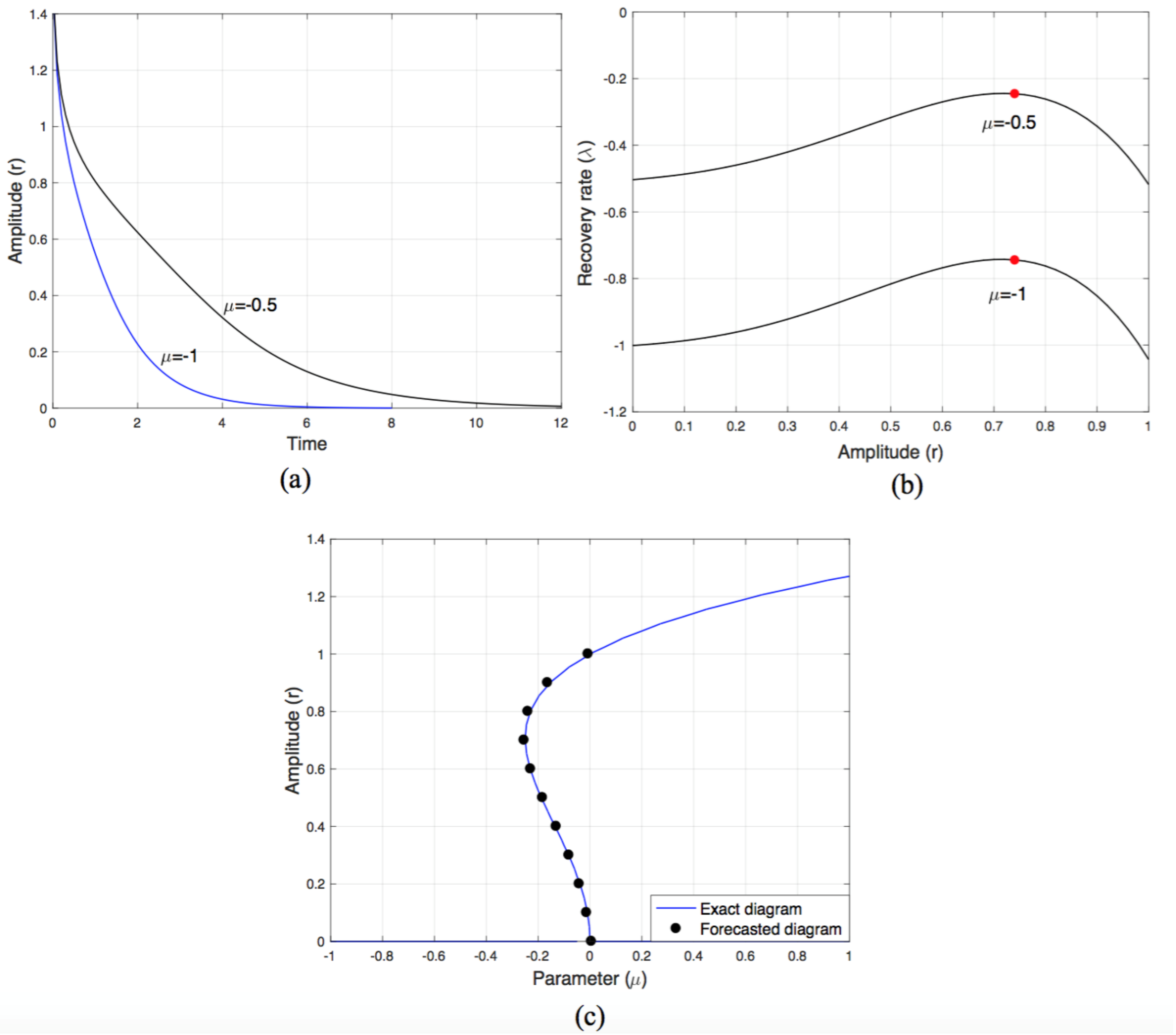

Fig. S7. Simulation results and forecasting procedure of "System 2" governed by Eq. (S6) and exhibiting a subcritical (catastrophic) transition.

5. Forecasting the bifurcation using the buddying yeast population model

To demonstrate the application of the proposed forecasting method when no measurement and no process noise is present, the method is evaluated theoretically using the simple population growth model (Eq. (S1)). The model parameters are approximated using genetic algorithm to fit best the experimental system (Table S1). The simulations are performed similarly to the

experimental procedure. We measure the simulated population every 23hrs, including the time lag. After each measurement, the selected dilution, which accounts for the mortality rate, is applied to the current population, and the new population density is simulated to grow for the next 23hrs. The model predicts that the population experiences a catastrophic transition at the dilution factor of 1,505. We forecast the bifurcation diagram assuming that the system approaches the transition from dilution factors first all larger, and then separately all smaller than the critical value. Thus, we examine separately the forecasting approach in the extinction and the emergence cases.

Consider a population which is approaching an extinction tipping point as the environmental conditions change. At a fixed dilution factor, the population recovers to its equilibrium after a perturbation is applied. The rate of recovery from perturbation depends on the distance to the tipping point. Figure S8(a) shows the system response to perturbations at two parameter values. Following the forecasting procedure, the recovery rates are computed, as demonstrated in Fig. S8(b). It is observed that the recovery rates at all amplitudes decrease as the system approaches the tipping point. Furthermore, the slowest speed of recovery is observed for the population amplitudes which are closest to the bifurcation diagram. In this example, the maximum recovery rate corresponds to the tipping point, which is the point on the bifurcation diagram that lies the furthest from the current parameter value (dilution factor 1,300 in this case). The forecasting results are presented in Fig. S8(c) which shows that the fixed points of the system are successfully forecasted. The tipping point is forecasted to occur at the dilution factor of 1,470. Importantly, it is evident from the results that the upcoming transition is catastrophic. Furthermore, one can identify the basins of attraction of the stable equilibria in the upcoming parameter values. For example, at the dilution factor of 1,415, it is forecasted that the minimum population density

at which the system maintains its ability to recover to the non-extinct state is $0.250\times10^{5} cells/\mu L$. A perturbation which would push the population below this boundary would lead to extinction.

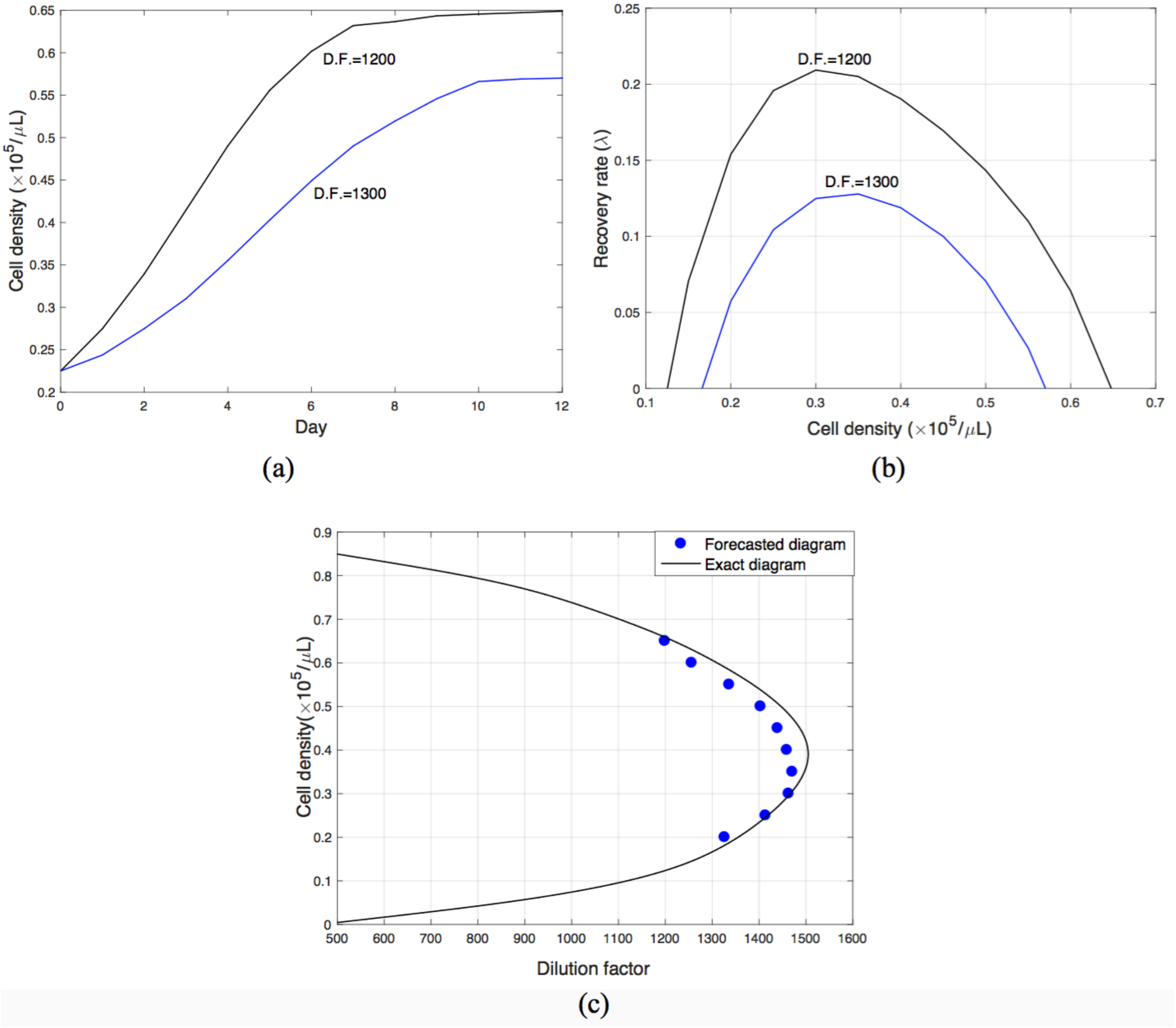

Fig. S8. Simulation results and forecasting of the bifurcation diagram using the budding yeast population model (Eq. (S1)) for the system approaching to the tipping point from dilution factors smaller than the critical value.

The same procedure is applied for the emergence case, when the system is approaching the bifurcation from dilution factors greater than 1,505. The system responses to large perturbations are demonstrated in Fig. S9(a), at three different dilution factors. One can observe from Fig. S9(b)

that at each dilution factor the recovery rate is fast at first, then it slows down, and after passing a specific population density it speeds up again. It is also shown that the slowest recovery rate corresponds to population values around $0.400\times10^5 cells/\mu L$ (as shown in Fig. S9(b)). Hence, the closest fixed point to the current state of the system has a non-zero amplitude which is a feature of subcritical (catastrophic) bifurcations. The forecasting results are shown in Fig. S9(c). The closest tipping point is forecasted correctly at the dilution factor of $1{,}470$, where the system enters to a bistable region and a large perturbation can push the system from one equilibrium to another. The forecasted bifurcation diagram shows that the system is approaching a catastrophic transition where a jump may occur between equilibrium points. The forecasted bifurcation diagram is in agreement with the diagram computed directly from Eq. (S1) (as shown in Fig. S9(c)).

It should be noted that the maximum forecasted amplitude in the bifurcation diagram cannot be greater than the maximum amplitude caused by perturbations. If, for example, the perturbations cause a change in cell density smaller than $0.400\times10^5\ cells/\mu L$, the tipping point cannot be forecasted. However, it can still be identified from the forecasted portion of the diagram that the upcoming transition is catastrophic.

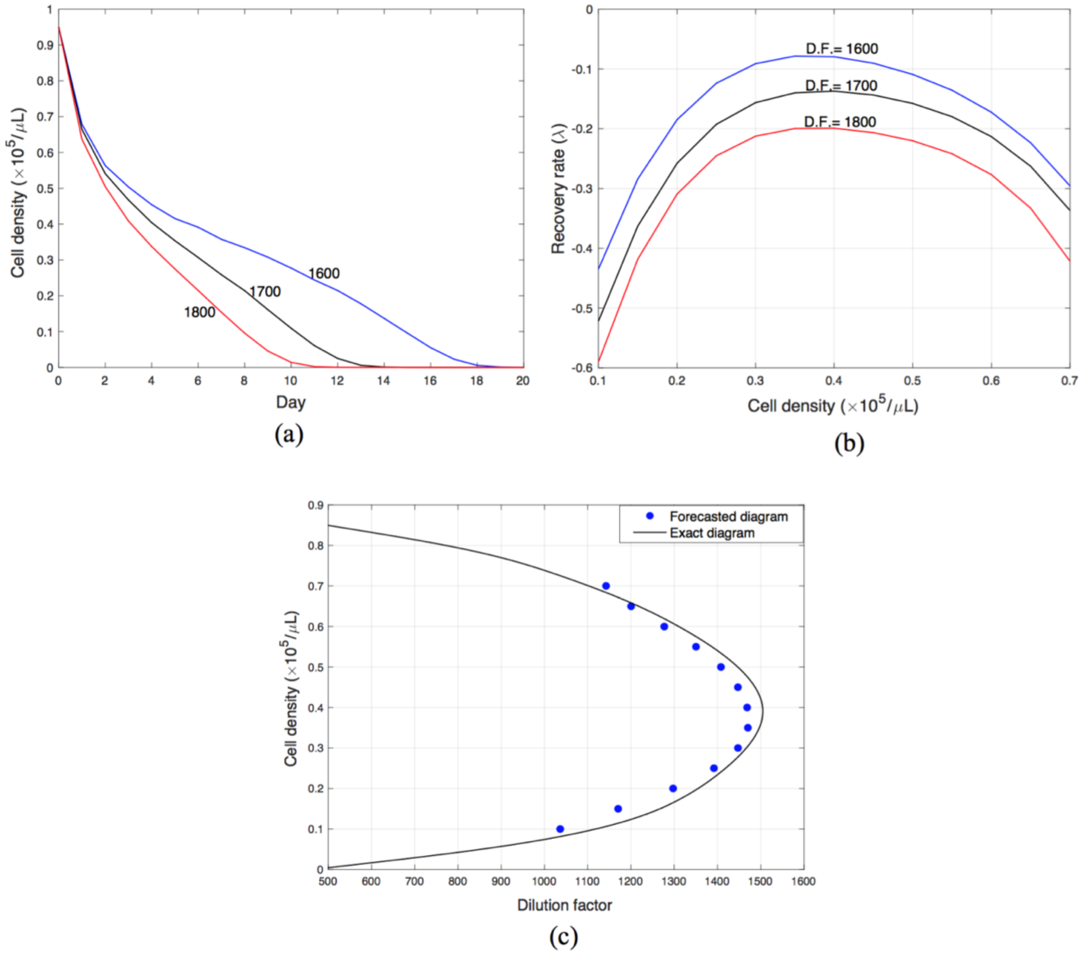

Fig. S9. Simulation results and forecasting of the bifurcation diagram using the budding yeast population model (Eq. (S1)), for the system approaching to the tipping point from dilution factors greater than the critical value.

These results show the applicability of the forecasting approach theoretically when no source of noise and uncertainty exists. In experimental applications, one needs more measurements to increase the accuracy and alleviate the effect of noise. However, the method is still expected to successfully forecast future stability of system, as is described in the main text.

References

1. Dai, L., Vorselen, D., Korolev, K. S. & Gore, J. Generic Indicators for Loss of Resilience Before a Tipping Point Leading to Population Collapse. *Science (80-.).* **336,** 1175–1177 (2012).
